# Metabarcoding avian diets at airports: implications for birdstrike hazard management planning

**DOI:** 10.1186/2041-2223-4-27

**Published:** 2013-12-11

**Authors:** Megan L Coghlan, Nicole E White, Dáithí C Murray, Jayne Houston, William Rutherford, Matthew I Bellgard, James Haile, Michael Bunce

**Affiliations:** 1 Australian Wildlife Forensic Services and Ancient DNA Laboratory, School of Veterinary and Life Sciences, Murdoch University, Murdoch, Western Australia 6150, Australia; 2 Ornithological Technical Services, Welshpool, Western Australia 6106, Australia; 3 Center for Comparative Genomics, Murdoch University, Murdoch, Western Australia 6150, Australia

**Keywords:** Birdstrike, Diet analysis, Species identification, Birdstrike management, Airport, Food chain

## Abstract

**Background:**

Wildlife collisions with aircraft cost the airline industry billions of dollars per annum and represent a public safety risk. Clearly, adapting aerodrome habitats to become less attractive to hazardous wildlife will reduce the incidence of collisions. Formulating effective habitat management strategies relies on accurate species identification of high-risk species. This can be successfully achieved for all strikes either through morphology and/or DNA-based identifications. Beyond species identification, dietary analysis of birdstrike gut contents can provide valuable intelligence for airport hazard management practices in regards to what food is attracting which species to aerodromes. Here, we present birdstrike identification and dietary data from Perth Airport, Western Australia, an aerodrome that saw approximately 140,000 aircraft movements in 2012. Next-generation high throughput DNA sequencing was employed to investigate 77 carcasses from 16 bird species collected over a 12-month period. Five DNA markers, which broadly characterize vertebrates, invertebrates and plants, were used to target three animal mitochondrial genes (12S rRNA, 16S rRNA, and COI) and a plastid gene (*trnL*) from DNA extracted from birdstrike carcass gastrointestinal tracts.

**Results:**

Over 151,000 DNA sequences were generated, filtered and analyzed by a fusion-tag amplicon sequencing approach. Across the 77 carcasses, the most commonly identified vertebrate was *Mus musculus* (house mouse). Acrididae (grasshoppers) was the most common invertebrate family identified, and Poaceae (grasses) the most commonly identified plant family. The DNA-based dietary data has the potential to provide some key insights into feeding ecologies within and around the aerodrome.

**Conclusions:**

The data generated here, together with the methodological approach, will greatly assist in the development of hazard management plans and, in combination with existing observational studies, provide an improved way to monitor the effectiveness of mitigation strategies (for example, netting of water, grass type, insecticides and so on) at aerodromes. It is hoped that with the insights provided by dietary data, airports will be able to allocate financial resources to the areas that will achieve the best outcomes for birdstrike reduction.

## Background

Bird and bat collisions with aircraft (henceforth referred to collectively as birdstrikes) are a significant issue for the aviation industry and in countries where reporting takes place, have been reported to be on the rise [1-3]. Growth in number of aircraft flight movements, as well as increases in aircraft size and airspeed, coupled with growing bird populations, are seen as leading factors in the increase of the incidence of birdstrikes [1,4]. The financial cost of a birdstrike can be substantial when considering direct damage to aircraft and loss of revenue during aircraft downtime [5,6]. Birdstrikes worldwide have been estimated to cost the commercial airline industry over one billion US dollars per year [7,8]. The first aviation fatality from a birdstrike was recorded in 1912 [9] and 100 years later, birdstrikes continue to be an ongoing health and safety concern. Since 1988, 221 people have been killed as a direct result of birdstrikes [10].

The USA reported approximately 9,730 birdstrike occurrences for the 2011 period alone [5,10]. While in Canada and the United Kingdom there were 1,513 (2009) and 2,457 (2011) birdstrikes reported respectively for a single year [11,12]. There were 1,758 birdstrikes within Australia during 2011, with a doubling of occurrences over the last decade for high capacity airports [13]. The overall worldwide trend is increasing, alongside improved reporting and documentation of birdstrike incidences.

**Figure 1:**
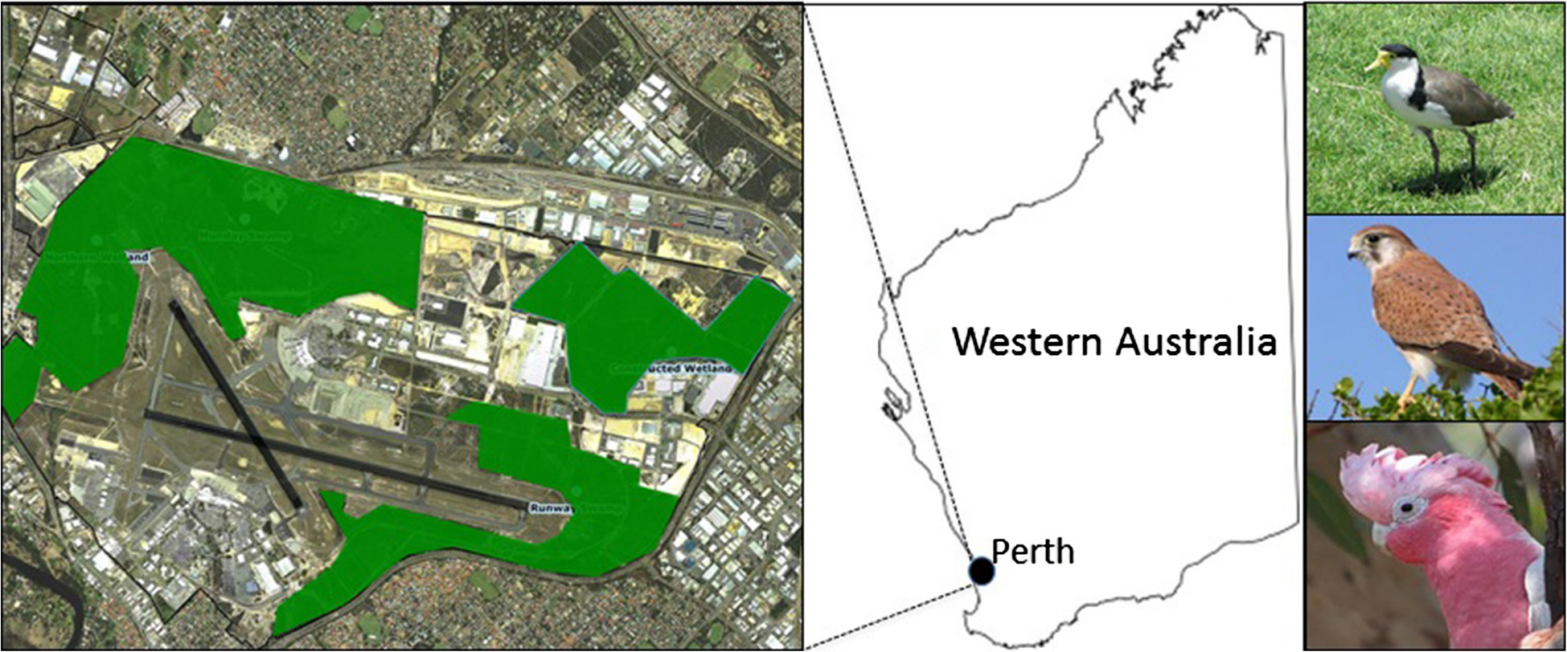
**The location and layout of the Perth Airport study site.** The aerial photograph (left) depicts both runways (in black) and high vegetation areas (highlighted in green). From top right to bottom right, the most common birdstrikes identified in Perth between 2002 and 2011: lapwing, Nankeen kestrel and galah.

Birdstrike mitigation strategies are used at all major aerodromes in an attempt to reduce the frequency of birdstrike occurrences. Some of these strategies include habitat modification, auditory and visual deterrents, avian radar systems, and changes to aircraft flight times and approach routes [10,13]. Some airports also specifically train their ground safety staff with the observational skills to accurately identify resident bird species, which is important in high-risk species. In the United States, the most commonly reported groups of birdstrike species causing the most damaging strikes were gulls, pigeons and doves, raptors, and waterfowl [10]. In the UK, geese and pigeons were identified as the most frequently struck bird species in 2008 [14]. In Australia, the most commonly hit animal between the years 2002 to 2011 were bats, followed by lapwings and plovers [13]. Figure 1 depicts the most commonly reported birdstrike species occurring in Perth, Western Australia, the focus site of this study. Establishing which species pose the highest risk for birdstrikes inevitably leads to more informed management plans for the reduction of incidences. Species identification of the remains of birdstrike victims is essential and can be made either by examining morphological characteristics or by the use of DNA analysis [1,3].

Morphological examination of feathers remains a primary identification technique for birdstrikes. However morphological analysis requires a high level of experience and correct identifications by morphology alone is difficult, either as a result of severe damage to the remains or to the inexperience of assessors, has been acknowledged in previous studies [1,15]. In cases where the quality of remains is too poor, DNA analysis may be the only viable method by which to make a species identification [1,3,6].

Dove, *et al*. [6], used a combination of both morphology and DNA barcoding using the mitochondrial cytochrome oxidase I (COI) gene to identify the bird species involved in a strike in Oklahoma, USA. This particular birdstrike caused a Cessna aircraft to crash, killing all five occupants in 2008. Both the morphological analysis of feather remains and DNA barcoding revealed that the bird species involved was the American white pelican (*Pelecanus erythrorhynchos*). Similarly, Marra, *et al*., [7] employed a combination of microscopic feather analysis and DNA barcoding to identify the Canada goose (*Branta canadensis*) as the cause of US Airways Flight 1549 ditching into the Hudson River in New York in January 2009.

Since most birdstrikes occur within a close proximity to aerodromes it is not only important to identify what species are involved, but it is equally important to determine why these species might be attracted to these areas. Analysis of diet using molecular techniques is an ideal method to determine what the main food sources are, and thus, why high-risk species are attracted to aerodrome environments. Additionally it may be possible to use dietary information as intelligence to develop management strategies to decrease or deter high-risk species habitation at aerodromes [16]. Thus where a main food source can be reduced, controlled or removed, a high-risk species may leave the area in search of more sustainable conditions [17].

Dietary analyses using next-generation DNA sequencing technology have been reported for species such as birds, bats, insects, and livestock [18-21]. In this study, for the first time, next-generation high-throughput DNA sequencing (HTS) is used to determine the major components of common birdstrike species diets. The aim of collating this data over a 12-month period is to provide empirical evidence of feeding habits and to integrate this information into best-practice management plans.

## Methods

### The Perth airport study site

Perth Airport (Figure 1) is located 10 km from the Perth central business district (CBD) on 2,105 ha of land that was historically used for farming and agriculture. Domestic and international terminals are situated centrally within the estate, with 306 ha of land reserved for conservation of unique flora and fauna, in the northern and southern precincts. There are four wetland areas on the estate, one in particular, Munday Swamp, is of high cultural significance to the traditional owners of this land, the Nyoongar people [22].

Surveys of the airport estate have shown that there are 134 different vertebrate species inhabiting the aerodrome. This number includes a minimum of 95 bird species, as well as native and introduced species such as the southern brown bandicoot and echidna, and house mouse, European rat, rabbits, foxes and feral cats. Endangered species such as the Carnaby’s black cockatoo (*Calyptorhynchus latirostris*) have been noted to be seasonally present at the aerodrome, as are red-tailed black cockatoos (*Calyptorhynchus banksii*), and feed on the Banksia and Marri within the conservation precincts [22].

### Birdstrike species identification through mitochondrial DNA sequencing

#### Use of animal samples

All specimens used in this study were deceased as a result of Perth Airport activities as governed by the bird and animal hazard management advisory committee (BAHMAC). Permission for the use of all birdstrike samples was granted by BAHMAC. No ethics permits were relevant in this study as researchers were using specimens that were otherwise scheduled for disposal.

#### Sample collection, DNA extraction, quantification and sequencing

Birdstrike remains (blood, tissue, and feathers) were collected from aircraft after arrival at Perth Airport by ground crew, safety officers and ornithologists. Airport personnel were instructed on how to collect biological remains, and were provided with DNA collection kits by Australian Wildlife Forensic Services (AWFS). The samples were transported to the AWFS lab for DNA extraction and species identification.

DNA extraction from blood swabs was carried out using a Qiagen QiaAmp DNA Micro kit (Qiagen, CA, USA) following the manufactures protocol. For feathers and tissue, samples were digested for 12 hours at 55°C in modified digest buffer (20 mM Tris (Sigma, MO, USA), 2.5 mM EDTA (Invitrogen, CA, USA), 5 mM CaCl_2_ (Sigma, MO, USA), 20 mM DTT (Thermo Fisher Scientific, MA, USA), 1% SDS (Invitrogen, CA, USA), 10 mg/mL Proteinase K (Amresco, OH, USA)) supernatant removed and mixed with PB buffer, bound to a Qiagen spin column (Qiagen, CA, USA), washed with AWI and AWII, and eluted from the column in EB buffer (Qiagen, CA, USA).

The DNA extracts were assessed for quantity and quality of mitochondrial DNA (mtDNA) using quantitative PCR (qPCR). This assessment allows for the identification of PCR inhibitors and low copy number DNA extracts. Three dilutions (undiluted, 1/10 and 1/100) for each DNA extract were assessed and the mitochondrial primers used were 12Sa and 12Sh (12S ribosomal RNA (rRNA)), [23] and MCB 398 and MCB 869 (Cytochrome b (Cytb)) [24]. Both mtDNA markers were amplified via qPCR in 25 μL volumes including: 2 mM MgCl_2_ (Fisher Biotec, Perth, Australia), 1 x Taq polymerase buffer (Fisher Biotec, Perth, Australia), 0.4 μM dNTPs (Astral Scientific, NSW, Australia), 0.1 mg bovine serum albumin (Fisher Biotec, Perth, Australia), 0.4 μM of each primer, and 0.2 μL of Taq DNA polymerase (AmpliTaq Gold, Applied Biosystems (ABI), CA, USA). The cycling conditions were: initial denaturation at 95°C for 5 minutes, followed by 50 cycles of 95°C for 30 seconds, annealing at primer specific temperature for 30 seconds, 72°C for 30 seconds, and a final extension at 72°C for 10 minutes (ABI, CA, USA). The annealing temperature used for 12S primers was 57°C and for Cytb was 51°C.

The above DNA extracts were amplified by PCR using the same 12S rRNA and Cytb primers following the same method described above for quantification, with 2 μL of template DNA at an optimal dilution as determined from the qPCR. The PCR products were then purified using a Qiagen PCR purification kit following the manufacture’s protocol, and were Sanger sequenced (Macrogen Inc, Seoul, Korea) in both directions for species identification using both mtDNA markers. The resulting sequences were imported into Geneious v4 [25], where BLASTn searches were carried out against the National Center for Biotechnology Information (NCBI) GenBank nucleotide NR database [26,27]. Species identifications were made based on the percentage similarity of the query sequence to the reference sequence (> 98%) as described previously [28]. For birdstrike species identifications, we chose to use a two gene method, rather than one, as we believe that this approach provides the best chance of making correct identifications based on current reference databases, as previously described [28]. We also chose to use 12S rRNA and Cytb over COI for species identifications because these gene regions, particularly Cytb, has been sequenced extensively in the past for studies of avian phylogenetics and as such, is one of the better represented genes on reference databases such as GenBank [29]. It is envisioned that with collection of more reference samples of bird species that are known to inhabit individual airports, reference databases will dramatically improve, including for COI gene regions, in the near future.

### Birdstrike species dietary analysis using HTS

#### Sample collection, DNA extraction and quantification

Seventy-seven bird carcasses were collected from Perth airport runways and surrounding areas by staff/contractors during the period of October 2011 to November 2012. Species identification was confirmed by morphological analysis by airport ornithologists. Birdstrike carcasses were stored in a −20°C freezer from the time the carcasses were found until transportation to the AWFS lab. The gastrointestinal tract (GIT) from the proventriculous/gizzard (stomach) through to the cloaca was dissected from each bird carcass. For the birds that sustained considerable body impact, all efforts were made to recover as much of the GIT as possible. A small portion of the contents from the stomach, small and large intestines were removed and collected into a 2 mL Eppendorf tube, to an approximate weight of between 100 and 900 mg depending on the size of the species and the volume within the GIT. This portion was used for subsequent DNA extraction. Dissected GITs and their contents were stored at −20°C prior to DNA extraction.

DNA was extracted from the GIT contents above using a modified QiaAmp DNA Stool Mini Kit method (Qiagen, CA, USA). The changes made to the kit protocol included an overnight digestion in buffer ASL at 55°C with the addition of 20 μL of Proteinase K after the first 30 minutes of the heat step. Additionally, only half of an InhibitEX tablet (Qiagen, CA, USA) was used in the protocol.

A broad, multi-locus approach was employed to examine what is likely to be a varied diet consumed by several bird species. Three mitochondrial genes (including two 16S rRNA primer sets; Additional file 1: Table S1) and a plastid gene were assessed using a range of universal primer sets. The primers used and which species were screened with each set are detailed in the Supplementary information, Additional file 1: Table S1. Firstly, the plant component of bird diet was examined by the use of a plastid gene marker, *trnL*. Some of the birdstrike species are known to consume small mammals, other birds, and fish, so the 16S rRNA gene was targeted for mammal and fish and the 12S rRNA region was targeted for detection of bird species. To analyze the insect content within the birdstrike species diet, the cytochrome oxidase I (COI) gene targeting insect species was used.

The extracted DNA was quantified by amplifying the following gene markers: *trn*L [30], 16S rRNA [19,31], 12S rRNA [23] and COI [32]. All qPCRs were carried out following the same PCR method as above for the birdstrike species identification samples.

#### Amplicon generation

Fusion primers with unique six to eight bp multiplex identifier (MID) tags were designed for the five primer sets (Additional file 1: Table S1) used for qPCR above, with unique forward and reverse combinations being allocated to each sample. The fusion primers also consisted of a GS FLX Titanium Primer A or B sequence preceding the MID tag [33]. The PCR protocols were carried out as for the qPCR above for each primer set. Amplicons were generated as previously described [20,34].

All fusion tagged PCR amplicons were double purified using the Agencourt AMPure XP Bead PCR purification protocol (Beckman Coulter Genomics, MA, USA). The purified PCR products were electrophoresed on a 2% agarose gel for 45 minutes together to confirm the presence of correct amplicon size and to determine a crude estimate of their concentration prior to equimolar pooling.

#### GS Junior sequencing set-up

Dilutions of the purified and pooled PCR amplicons were made and quantified using a synthetic oligonucleotide standard of known molarity [35], to approximate an optimal bead to template ratio for emulsion PCR. Once this ratio was determined, the emulsion PCR, bead recovery and sequencing steps for the Roche GS Junior (CT, USA) were carried out in accordance with Roche protocols.

#### Analysis of GS Junior sequencing data

The sequencing output FASTA (.fna) files were retrieved in their raw form and processed using the following protocols. The FASTA files were imported into the program Geneious v6.1.3 [25], for deconvolution. Sequences with 100% exact adaptors and MID tags were identified and sorted. The annotated and filtered sequences were exported as fasta files and imported into YABI [36] where a BLASTn search was conducted against the NCBI GenBank nucleotide NR database [26,27]. The resultant BLAST files were then imported into the program MEtaGenome ANalyzer v4.69.4 (MEGAN) [37] for examination of taxonomic assignment. The amplicon sequences were assigned using the following lowest common ancestor parameters: minimum score of 65, top percent of 5, and minimum support of 1. As discussed previously [34,38] assignment fidelity for plants is largely dependent on the coverage of the NCBI reference database and the robustness of the underpinning taxonomic framework.

## Results and discussion

Our initial work with Perth Airport began with providing mtDNA species identifications of biological remains where morphological identification was not possible (Table 1). This developed into a more fundamental question regarding what was attracting bird species to the aerodrome in the first instance. While there are morphological means of determining diet [16] there is a push towards molecular systems due to benefits with accuracy and sensitivity. The mtDNA species identifications of biological remains taken from aircraft and the dietary analysis of bird carcasses are discussed separately below.

**Table 1:** Species identification of birdstrike remains (blood, tissue, feathers) collected from aircraft after landing at Perth Airport

**Case number**	**Sample type**	**Species identified**	**Common name**	**Comments**
1	Blood soaked feather	*Bubulcus ibis*	Cattle egret	100% match 12S and Cytb
2	Blood swab	*Columba livia*	Common pigeon	100% match 12S only
3	Feather	*Columba livia*	Common pigeon	100% match 12S only
4a	Blood swab	*Fulica atra australis*	Australian coot	100% match 12S only
4b	Feather	*Fulica atra australis*	Australian coot	100% match 12S only
5	Blood swab	Hirundinidae	Swallow and martin family	97% match with 12S
		*Petrochelidon ariel*	Fairy martin	98% match with Cytb
6	Partial carcass	*Chroicocephalus hartlaubii*	Hartlaub’s gull*	100% match Cytb only
		*Chroicocephalus cirrocephalus*	Grey-headed gull*	100% match Cytb only
7	Blood swab	N/A	N/A	Extract failed to amplify DNA
8	Feather	*Apus pacificus*	Pacific swift	100% match 12S and Cytb
9	Blood swab	Hirundinidae	Swallow and martin family	97.9% match with 12S
		*Hirundo nigricans*	Tree martin	99.8% match with Cytb
10	Feather	*Falco berigora*	Brown falcon	100% match Cytb only. Strike reported on take off
11	Blood swab	*Pteropus poliocephalus*	Grey‒headed flying fox	99.4% match with Cytb. Strike occurred on take off
12	Blood swab	Accipitridae	Birds of prey	90.5% match 12S, 90.9% match Cytb
13a	Blood swab	*Chenonetta jubata*	Australian wood duck	90.5% match 12S, 90.9% match Cytb
13b	Blood swab	*Chenonetta jubata*	Australian wood duck	90.5% match 12S, 90.9% match Cytb

Species identification by the Australian Wildife Forensic Services used mitochondrial DNA sequencing (12S rRNA and/or Cytb).

* Cytb sequences matched 100% to two *Chroicocephalus* species.

### Species identification of biological remains

AWFS assisted Perth Airport with 13 separate birdstrike incidents during the period 2010 to present. In 12 out of 13 cases, DNA identifications were successfully made (Table 1) via the sequencing of mtDNA gene regions (12S and/or Cytb). Identifications were made to the species level in eight of the cases (case numbers 1, 2, 3, 4, 8, 10, 11, and 13), to genus level in three cases (case numbers 5, 6, and 9), and to the family level only in one case (case number 12; See Table 1). The species identified included cattle egret, feral pigeon, Australian coot, Pacific swift, brown falcon, grey-headed flying fox, and Australian wood duck. In eight cases, identifications were made using one of the two mtDNA genes, 12S or Cytb, only. In case number six, the identification was made to genus level using Cytb, however, the taxonomic resolution for this marker was not sufficient to make a species level identification, as at least two species within the genus *Chroicocephalus* had identical sequences for this marker. A lack of reference sequences within the database (GenBank) for the collected birdstrike species is apparent due to the inability to make species level identifications in four of the cases. The importance of using two genes is exemplified here, and the fact that one gene does not appear to be better than the other in terms of frequency in making identifications, shows that a two gene approach is more successful than choosing one gene for this purpose.

While species identification of birdstrike remains is valuable for airports for various reasons, one being the detection of high-risk species, this however, does not provide insight into why these species were at the aerodrome and whether habitat surrounding the site was a contributing factor in the strike. Determining the major diet components of high-risk species will provide answers as to why airports are attractive habitats.

### Dietary analysis of bird GIT contents

Next generation DNA sequencing was used to identify the GIT contents of 77 birds comprising 16 different species. Twelve out of 16 bird species contained animal and/or insect DNA within their GIT contents, ranging across eight animal classes, from Mammalia to Actinopterygii (Table 2). Plant DNA was identified in all 16 species, ranging across 24 plant families (Table 3). The findings are presented and discussed below.

**Table 2:** Closest animal DNA sequence matches generated through high-throughput sequencing (HTS) from within the gastrointestinal tracts (GITs) of 12 birdstrike species collected at Perth Airport

**Birdstrike species**	**Animal classes, orders, families, genera and species identified**										
	***Mus musculus***	***Bos***	***Zosterops lateralis***	**Scincidae**	***Gambusia***	**Aranea**	**Acrididae**	**Lepidoptera**	**Diptera**	***Rhantus***	***Cherax***
Australian hobby (*n* = 1)			✓				✓				
Barn owl (*n* = 9)	✓							✓			
Nankeen kestrel (*n* = 16)	✓			✓			✓	✓	✓		
Black shouldered kite (*n* = 1)	✓										
Southern boobook (*n* = 1*)*	✓							✓			
Australian Magpie (*n* = 1)		✓									
Galah (*n* = 13)									✓		
White-faced heron (*n* = 8)					✓	✓	✓		✓		✓
Pacific black duck (*n* = 1)									✓	✓	
Welcome swallow (*n* = 3)									✓		
Little button quail (*n* = 1)							✓				
Pipit (*n* = 3)							✓				

**Table 3:** Plant DNA sequence matches generated from high-throughput sequencing (HTS) of gastrointestinal tract (GIT) contents of 16 birdstrike species collected at Perth Airport

**Plant families and genera identified**		**Birdstrike species**															
**Plant family**	**Plant genus**	**AH**	**BO**	**NK**	**BSK**	**SB**	**AM**	**RTBC**	**G**	**WFH**	**WD**	**PBD**	**P**	**TM**	**WS**	**LBQ**	**Pip**
**Aizoaceae**									✓								
**Amaranthaceae**				✓										✓	✓		
**Apiaceae**		✓															
**Asteraceae**				✓			✓		✓	✓				✓	✓		
**Brassicaceae**													✓	✓			
**Cyperaceae**										✓							
**Fabaceae**			✓	✓													
	*Mirbelieae*					✓											
	*Trifolium*		✓							✓	✓						
**Geraniaceae**	*Erodium*								✓								
**Haloragaceae**	*Myriophyllum*						✓										
**Iridaceae**			✓	✓			✓		✓	✓							✓
**Juncaceae**	*Juncus*											✓					
**Lamiaceae**	*Salvia*						✓										
**Myrtaceae**						✓	✓	✓	✓					✓			
**Oleaceae**			✓														
**Oxalidaceae**	*Oxalis*		✓						✓	✓							✓
**Pittosporaceae**	*Billardiera*						✓										
**Plantaginaceae**	*Callitriche*										✓						
**Poaceae**			✓	✓	✓		✓		✓	✓	✓	✓	✓			✓	✓
	*Avena*		✓						✓				✓				
	*Phalaris*												✓				
	*Sorghum*												✓				
**Polygonaceae**				✓										✓			
**Proteaceae**			✓														
**Rosaceae**	*Rosa*						✓										
**Solanaceae**		✓	✓	✓						✓				✓	✓		
**Verbenaceae**							✓										
**Vitaceae**														✓			
**Number of individuals sampled**		1	9	16	1	1	2	1	13	8	2	1	8	7	3	1	3

Birdstrike species name abbreviations: AH, Australian hobby; BO, barn owl; NK, Nankeen kestrel; BSK, black-shouldered kite; SB, southern boobook; AM, Australian magpie; RTBC, red-tailed black cockatoo; G, galah; WFH, white-faced heron; WD, wood duck; PBD, Pacific black duck; P, pigeon; TM, tree martin; WS, welcome swallow; LBQ, little button quail; Pip, pipit.

#### Animal and insect DNA identified

Overall, 16S rRNA and 12S rRNA data targeting mammal, bird and fish prey was generated from seven bird species. Our results show that four of these were identified to have *Mus musculus* (house mouse) (Table 2), within their GIT contents. All four of these species are raptors, which are known to consume small mammals such as mice, as part of their diet. Feral mice are present in large quantities all over Australia and their presence is not unexpected given the food waste that is generated daily at airports. The GIT contents of the Australian hobby (*Falco longipennis*), did not contain *M. musculus,* however, there were DNA sequences matching *Zosterops lateralis,* a small passerine bird, detected when this sample was screened with 12S rRNA bird specific primers. Australian hobbys are known to consume other small birds and insects as part of their diet [39]. A species of *Bos* (Bovidae) was detected within the GIT of one magpie sample. It is possible that this result is due to direct scavenging on food waste either at or immediately surrounding the aerodrome. Management strategies that aim to reduce available food sources for rodents and scavenging animals include the strict isolation and control of food waste and rubbish bins at aerodromes [40].

Two white-faced herons were found to contain DNA closely matching mosquito fish (genus *Gambusia*; 98% match using 16S rRNA; Table 2) within the GIT, while another heron contained DNA closely matching freshwater crayfish (genus *Cherax*; 99% match using COI primer set; Table 2). Two white-faced herons also were consuming a species of spider (order Araneae), as well as insect species within the order Diptera. The diet of white-faced herons is known to be widely variable, and opportunistic depending on the season, and includes the types of aquatic species identified here, as well as other insects, frogs and reptiles [41]. There are several drains and wetlands at Perth airport, which likely contain the aquatic species identified in this study. Mosquito fish were previously noted to be present at the airport estate after an aerodrome survey. It is possible that direct control of invasive species (for example, rotenone to inhibit feral fish) might reduce the prevalence of these prey species, but as always, such control needs to be balanced against the effect this would have on native species. Many aerodromes worldwide use management policies for ponds that include netting, converting above ground drainways to underground, and improving drainage of rain water from the site [40,42].

Of ten birdstrike species screened with insect COI primers, 50% were found to be consuming a species of Acrididae (grasshoppers). The closest BLAST matches that were identified were from two Acrididae species, *Prumna arctica* (95% to 96%) and *Sphingonotus tsinlingensis* (94.9%), which are not likely to be the species in question due to the low percentage similarity of the sequences. Faunal surveys of Perth Airport have identified the presence of Australian plague locust (*Chortoicetes terminifera*) in high abundance, and their presence has been an ongoing problem for the aerodrome. The abundance of this species at Perth Airport, and visual detection of locust in the GIT contents of the birdstrike species during dissection, means the identification can be reasonably made through association. Spraying of the fungicide, Green Guard (Yates, NSW, Australia), to control locust populations has been trialed to attempt to reduce the *C. terminifera* populations at the aerodrome. So far, this has been shown to have a positive effect in reducing the number of locusts where spraying occurred. Locusts are known to be seasonally variable and feed on grasses and crops. The abundance of Acrididae species in the diets of birdstrike species points towards the need for more active control strategies.

#### Plant diet

Twenty-four plant families were identified within the GIT contents of all 16 birdstrike species. The most common plant Family identified was Poaceae (grasses), with this Family present in 11 species (Table 3). The next most common plant Families identified were: Asteraceae, Iridaceae, and Solanaceae (Table 3). Species from the Family Poaceae appear to be a significant diet component of either the birdstrike species directly, or of their prey species. It is prudent to point out here that in the current study it is not possible to differentiate where some of the identified plant species fit in the bird species food chain. Species of Poaceae would likely comprise a major component of the diet of the Acrididae and mice species that are consumed by the birdstrike species, thus being a secondary item in the food chain. Whether or not Poaceae is a first or secondary food item for birdstrike species, it is likely to be a significant component of the prey diet and should still remain a management priority for airports.

Management of grass has been recognized as being of high importance at airports as a birdstrike mitigation strategy for some time. Airports in the UK use a long grass policy in habitat management plans. Short grass has been shown to encourage birds by providing the best environment for invertebrates, which in turn encourages birds to search for food here. Short grass also provides birds with a better view of the environment, giving increased security for feeding. Maintaining grass at a length of at least 15 to 20 cm is ideal in the UK for limiting the ability of birds to find and feed on invertebrates, and reduces the availability of cleared, safe feeding zones [40]. At Perth Airport, the grass length is maintained at a length up to 30 cm. Grasses that have been fungal-endophyte enhanced, such as AVANEX® (PGG Wrightson Seeds, Victoria, Australia) developed and trialed in New Zealand, have provided successful reduction in insect and bird numbers where the grass has been installed [43,44]. Interestingly, trials of grass length and the growth of less attractive/palatable species could be assessed in a cost-effective manner using HTS methods and this is likely a far better proxy for palatability than use of a simple bird count survey and/or frequency of birdstrikes.

For obvious reasons, flocking birds represent the greatest threat to aircraft. At Perth airport the galah (*Eolophus roseicapilla*) weighing in at approximately 300 g represents one high-risk species. Galahs usually forage on the ground, searching for and ingesting seeds which are located by sight, and it has long been suspected that the weed common storksbill (*Erodium* sp.) at the aerodrome was the target. The weed is attractive to galahs during the hatching season (September) when it is green and easily digestible. Galahs were the only species in this study to have been found to have *Erodium* sp. identified within the GIT. An *Erodium* control program has been in place at Perth Airport for the last five years, and during this time it has been observed that galahs are spending less time within the airport estate. Clearly, on the basis of this empirical data *Erodium* control should be a priority at specific times of the year.

Although identifying prey species is important, the strength of such a method will come from continued monitoring of diet to assess the effectiveness of habitat management plans that have been put into place. Beyond comparing the total number of strikes that occur, it would be practical to assess how, or if, diets of high-risk species change, and the distributions of prey species populations within the aerodrome post management implementation. An extension to this study might be to investigate mice GITs or fecal material to develop a more holistic insight into food webs at the airport. It is possible that certain insect and grass species identified in this study could be the prey of mice or Acrididae species, rather than the birdstrike species, but would need to be assessed further to determine this, for which, a similar HTS method could be employed on these commonly consumed birdstrike prey species.

## Conclusions

The interaction of volant wildlife and aircraft is unavoidable; however, it is possible to make aerodromes less attractive from the perspective of food availability. Based on the dietary food chain data generated in this study, several key areas of focus could be developed to enhance existing habitat management strategies. The key components of the birdstrike species (and/or prey species) diets to highlight as priorities included management of grass (height and species) and *Erodium* sp., the use of fungicides/insecticides to decrease the population of Acrididae, rodent control, and a water management strategy for wetlands and drains.

The advantages of dietary analyses of birdstrike carcasses are not limited to assisting in airport hazard management planning. Ecological and species population data could also be studied from this sample resource. Some birdstrike species may be of interest from a conservation perspective, for example one of the birds in this dataset was the red-tailed black cockatoo (*Calyptorhynchus banksii*) of which little is known about its diet especially in urban environments. In this situation, the opportunity to study the diet of such species makes best scientific use of a sample that is otherwise discarded and is difficult to procure through other means. Airports that can allocate resources to the collection of birdstrike carcasses, such as Perth, are helping to ensure that this type of research can occur.

Analysis of the plant and animal components of common birdstrike species diets using HTS is an extremely valuable tool. Sequencing platforms, such as 454 (Roche, CT, USA), Ion Torrent (Life Technologies, CA, USA) and MiSeq (Illumina, CA, USA) can provide a large amount of sequence data within a short time frame. Identifying the most prominent food sources within the aerodrome environment for high-risk bird species (and possibly their prey) can help to plan tailored, best-practice hazard management strategies in an effort to reduce wildlife strikes, and help airports to partition financial resources most efficiently. Using effective management plans to lower birdstrike frequency would assist in decreasing the burdensome cost of aircraft repairs, flight rescheduling, and passenger safety risk, which thus highlights the applied nature of DNA-based food chain studies in complex environments such as aerodromes.

## Abbreviations

BLAST: Basic local alignment search tool; Bp: Base pair; CBD: Central business district; Cytb: Cytochrome b; COI: Cytochrome oxidase subunit I; GIT: Gastrointestinal tract; HTS: High-throughput sequencing; AWFS: Australian wildlife forensic services; qPCR: Quantitative polymerase chain reaction; MID: Multiplex identifier; MEGAN: MEtaGenome aNalyzer; mtDNA: Mitochondrial DNA.

## Competing interests

The authors declare that they have no competing interests.

## Author’s contributions

Conceived and designed the experiments: MLC and MB. Performed the experiments: MLC, NEW and JH. Collected the bird carcasses and provided morphological species identifications: WR. Analyzed the data: MLC, DCM, NEW, MB, and MIB. Wrote the paper: MLC, JH, NEW, and MB. All authors read and approved the final manuscript.

## Supplementary Material

Additional file 1: Table S1 List of primers used in this study for generating fusion tagged amplicons for high-throughput sequencing (HTS). Click here for file
